# Nonspiro, Fluorene‐Based, Amorphous Hole Transporting Materials for Efficient and Stable Perovskite Solar Cells

**DOI:** 10.1002/advs.201700811

**Published:** 2018-01-31

**Authors:** Šarūnė Daškevičiū tė, Nobuya Sakai, Marius Franckevičius, Marytė Daškevičienė, Artiom Magomedov, Vygintas Jankauskas, Henry J. Snaith, Vytautas Getautis

**Affiliations:** ^1^ Department of Organic Chemistry Kaunas University of Technology Radvilenu˛ pl. 19 Kaunas LT‐50254 Lithuania; ^2^ Department of Physics Clarendon Laboratory University of Oxford Parks Road Oxford OX1 3PU UK; ^3^ Center for Physical Sciences and Technology Saulėtekio Ave. 3 Vilnius LT‐10257 Lithuania; ^4^ Institute of Chemical Physics Vilnius University Saulėtekio al.3 Vilnius LT‐10257 Lithuania

**Keywords:** fluorene, materials science, organic conductor, perovskite, solar cells

## Abstract

Novel nonspiro, fluorene‐based, small‐molecule hole transporting materials (HTMs) **V1050** and **V1061** are designed and synthesized using a facile three‐step synthetic route. The synthesized compounds exhibit amorphous nature with a high glass transition temperature, a good solubility, and decent thermal stability. The planar perovskite solar cells (PSCs) employing **V1050** generated an excellent power conversion efficiency of 18.3%, which is comparable to 18.9% obtained with the state‐of‐the‐art Spiro‐OMeTAD. Importantly, the devices based on **V1050** and **V1061** show better stability compared to devices based on Spiro‐OMeTAD when aged without any encapsulation under uncontrolled humidity conditions (relative humidity around 60%) in the dark and under continuous full sun illumination.

## 1 Introduction

Due to the merits of intense absorption in an almost entire visible spectral region, perovskite materials first emerged as dye substitutes for dye‐sensitized solar cells with the liquid electrolyte showing a power conversion efficiency (PCE) around 4%.^1^ Since the first perovskite‐based solar cells (PSCs) suffered from fast degradation their architecture was shifted from liquid electrolyte‐based low PCE devices to solid‐state devices using small organic hole transporting molecule 2,2′,7,7′‐tetrakis(*N,N*‐di‐*p*‐methoxy‐phenylamine)‐9,9′‐spirobifluorene (Spiro‐OMeTAD).^2^ In consequence, the photovoltaic performances have skyrocketed to 21.1% for small‐area cells^3^ and 20.5% for cells^4^ with the active area larger than 1 cm^2^ in just a few years. Since then, Spiro‐OMeTAD became a standard material for the development of the new hole transporting materials (HTMs) for PSCs. In the majority of the state‐of‐the‐art devices, Spiro‐OMeTAD is used as a hole transporting material.^5^, ^6^, ^7^, ^8^ However, the tedious synthetic procedures and purification processes of this HTM make it cost ineffective and thus limit its application and commercialization.^9^ Moreover, the study by A. Binek et al. showed that Spiro‐OMeTAD can significantly contribute to the overall cost of materials required for the PSC manufacturing.^10^

To engineer HTMs which are considerably cheaper than Spiro‐OMeTAD, shorter reaction schemes with simple purification procedures are required. Successful examples include azomethine derivative EDOT‐OMeTPA,^11^ branched methoxydiphenylamine‐substituted fluorene derivatives V859 and V862,^12^ enamine derivative V950,^13^ and spiro[fluorene‐9,9′‐xanthene] (SFX)‐based materials SFXMeOTAD^14^ or X60,^15^ and X59.^16^ Some of them are reported to have equal or slightly better performance in comparison to that of Spiro‐OMeTAD.^12^, ^15^

Besides the low cost, HTMs should meet a number of other requirements, including excellent charge transporting properties, good energy matching with the perovskite, transparency to solar radiation, large Stokes shift, good solubility in organic solvents, morphologically stable film formation, and others. Meanwhile, numerous investigations are being carried out aiming to improve the efficiency of HTMs, however until now only few concrete recommendations have been made with regard to the molecular structure modification.^17^, ^18^, ^19^, ^20^, ^21^, ^22^, ^23^, ^24^

Recently, we have presented the synthesis of a new class of efficient HTMs.^25^, ^26^ Their structure consists of two 4,4′‐dimethoxydiphenylamine 3,6‐disubstituted carbazole fragments linked by a nonplanar unit. Synthesis of these HTMs was performed by a simple two‐step synthetic procedure providing a target product in high yield. Conveniently, the first step is a basic “click” type reaction and high reaction rates (≈10 min at room temperature), simple purification procedure (only filtration is required) and high yields are typical for this step. The performance of CH_3_NH_3_PbI_3_‐based PSC with these branched compounds was close to that of the Spiro‐OMeTAD in the same conditions.

**Figure 1:**
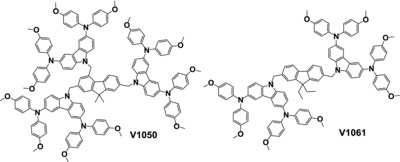
Structures of HTMs: **V1050** and **V1061**.

In this work, we present the synthesis, characterization and photovoltaic performance of two new hole transporting materials, namely **V1050** and **V1061** possessing 4,4′‐dimethoxydiphenylamine 3,6‐disubstituted carbazole‐based hole transporting moieties and 9,9‐dialkyl‐9*H*‐fluorene as a central linking fragment (**Figure** **1**).

The coplanar central core was chosen in order to improve the efficiency of HTM, as was previously shown by Li and co‐workers.^17^ Furthermore, the properties and performance of newly synthesized HTMs were additionally compared to that of the state‐of‐the‐art Spiro‐OMeTAD.

## 2 Results and Discussions

### 2.1 Synthesis

**Scheme 1:**
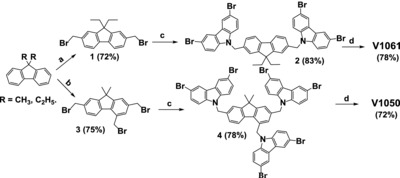
Synthesis route to HTMs **V1050** and **V1061**: a) paraformaldehyde (2.2 equiv.), 33% HBr in HOAc, 65 –70 °C; b) paraformaldehyde (10.0 equiv), 33% HBr in HOAc, 85 –90 °C; c) 3,6‐dibromocarbazole, 85% KOH, THF, at r.t.; d) 4,4′‐dimethoxydiphenylamine, Pd(OAc)_2_, P(*t*‐Bu)_3_·BF_3_, NaO*t*‐Bu, at reflux of anhydrous toluene.

The fluorene derivatives **V1050** and **V1061** were synthesized by a simple two‐step reaction from the key intermediates, the bromomethylfluorenes **1** and **3**, as illustrated in **Scheme** **1**. To synthesize intermediate **3**, a slightly modified literature procedure was employed to perform the tris(bromomethylation) of 9,9‐dimethylfluorene.^27^ In the next step, 3,6‐dibromo‐9*H*‐carbazole was reacted with 2,4,7‐tris(bromomethyl)‐9,9‐dimethyl‐9*H*‐fluorene (**3**) in the presence of KOH powder to provide the intermediate compound **4**. Finally, the desired product **V1050** was obtained by the palladium‐catalyzed Buchwald–Hartwig C–N cross‐coupling reaction of compound **4** with 4,4′‐dimethoxydiphenylamine. Surprisingly, di(bromomethylation) of 9,9‐dimethyl‐9*H*‐fluorene was unsuccessful and inseparable mixture of the reaction products was obtained by using two equivalents of paraformaldehyde. Presumably, the presence of the small methyl substituents at the 9th position markedly stipulates the formation of trisubstituted product as well. Therefore, as the reference compound, the hole transporting material **V1061**, containing 9,9‐diethyl‐9*H*‐fluorene as a central linking fragment and two equivalent 4,4′‐dimethoxydiphenylamine 3,6‐disubstituted carbazole‐based branches was also prepared according to the same approach. The final compounds **V1050** and **V1061** were isolated by column chromatography and precipitated from toluene or tetrahydrofuran into 15‐fold excess of *n*‐hexane. Obtained by such a procedure **V1050** and **V1061** were amorphous compounds. All our attempts to crystallize them were unsuccessful. The chemical structure of the synthesized **V1050** and **V1061** products were confirmed by ^1^H and ^13^C NMR as well as elemental analysis data. A more detailed procedure for the synthesis of hole transport materials is included in the Supporting Information.

### 2.2 Thermal Properties

**Figure 2:**
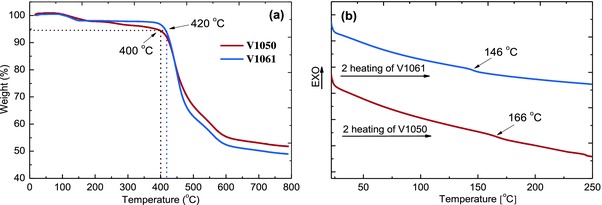
a) TGA curves of the **V1050** and **V1061** (heating rate 10 °C min^−1^), b) DSC second heating curves for the **V1050** and **V1061** (heating rate 10 °C min^−1^).

Thermal gravimetric analysis (TGA) and differential scanning calorimetry (DSC) were applied to measure the thermal stability of the above obtained HTMs. The weight loss as a function of temperature of **V1050** and **V1061** compounds is presented in **Figure** **2**a. A significant weight loss appears at around 400 °C and proceeds until complete decomposition of both materials at 600 °C.

The decomposition temperature corresponding to a 5% weight loss (*T*_d_) is about 400 °C, indicating good thermal stability of **V1050**. HTM possessing two 4,4′‐dimethoxydiphenylamine 3,6‐disubstituted carbazole‐based branches shows slightly better thermal stability because *T*_d_ is about 420 °C. From TGA measurements we conclude, that both fluorene‐based compounds are suitable for the application in the perovskite solar cells. Initial small (2–3%) weight loss at <150 °C can be attributed to the evaporation of the solvent traces remaining after the purification procedure.

DSC analysis of the **V1050** and **V1061** has shown (Figure 2b) that after purification, the target compounds exist in an amorphous state with the glass transition temperatures of about 166 and 146 °C, respectively. The high glass transition temperature is in good agreement with one of Wirth postulates^28^ demonstrating that the glass transition temperature can be raised by increasing molecular size, incorporating additional bulky substituent into 4th position of the molecule, and enhancing molecular interaction, which can hinder molecular motions. To the best of our knowledge, **V1050** exhibits one of the highest glass transition temperatures among amorphous HTMs applied in the PSCs, being 40 °C higher than that of Spiro‐OMeTAD (126 °C). A high glass transition temperature is an indicator of the more stable amorphous state and reduced tendency to crystallize. Recently, it has been observed that Spiro‐OMeTAD tends to crystallize in perovskite solar cells under device operating conditions,^29^ which in turn can lead to device degradation and failure over the longer term. Whereas, both **V1050** and **V1061** are entirely amorphous and do not show characteristic transitions of the crystalline state (Figures S1 and S2, Supporting Information). The superior properties of new HTMs can be attributed to the presence of the branched 4,4′‐dimethoxydiphenylamine 3,6‐disubstituted carbazole‐based moieties linked by bulky central fluorene core.

### 2.3 Optical and Photophysical Properties

**Figure 3:**
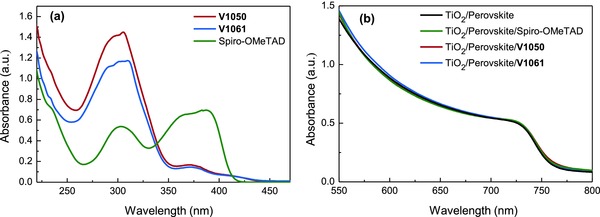
a) UV–vis absorption spectra of **V1050**, **V1061**, and Spiro‐OMeTAD, b) UV–vis absorption spectra of TiO_2_/perovskite (FA_0.83_Cs_0.17_Pb(I_0.8_Br_0.2_)_3_) film and perovskite (FA_0.83_Cs_0.17_Pb(I_0.8_Br_0.2_)_3_) deposited with Spiro‐OMeTAD and **V1050**, and **V1061** hole transporting materials.

The UV–vis absorption spectra of a new fluorene‐based organic HTMs measured in tetrahydrofuran (THF) solution is shown in **Figure** **3**a and compared with Spiro‐OMeTAD. The absorption spectra for both **V1050** and **V1061** HTMs are almost identical. This confirms that conjugation between 4,4′‐dimethoxydiphenylamine 3,6‐disubstituted carbazole‐based branches is absent. Both HTMs show intense π–π* absorption band with the maximum at 305 nm and weak low energy absorption at 375 nm corresponding to n–π* transitions. Furthermore, absorption of both compounds lies mainly in the UV region with a very weak undesirable absorption in the visible spectral range. Because a new hole transporting materials are more transparent to solar radiation, they become even more advantageous for the application in the PSC.^30^ The steady state absorption spectra of perovskite FA_0.83_Cs_0.17_Pb(I_0.8_Br_0.2_)_3_ films deposited on TiO_2_ with and without hole transporting materials are shown in Figure 3b. The absorption spectra show characteristic absorption onset at around 775 nm due to exciton absorption. All films reveal very similar signatures when measured above 550 nm, indicating that there is no apparent contribution of HTMs to the light absorption of perovskite solar cells.

**Figure 4:**
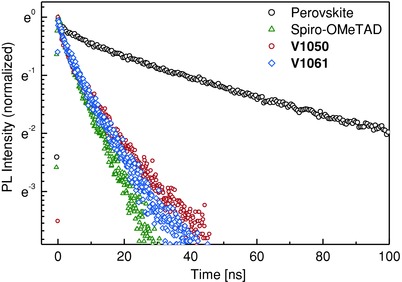
Photoluminescence decay kinetics of lead halide perovskite (FA_0.83_Cs_0.17_Pb(I_0.8_Br_0.2_)_3_) films deposited on glass substrates comprising **V1050**, **V1061**, and Spiro‐OMeTAD hole transporting layers. Photoluminescence lifetimes were monitored at the emission maximum at 770 nm upon excitation at 470 nm.

Here, we additionally performed time resolved photoluminescence (PL) decay measurements to study charge–carrier transport properties in perovskite films deposited on glass with and without HTMs (**Figure** **4**). For pristine perovskite films, PL decays during 58 ns and reflects nongeminate electron–hole recombination^30^ being the dominating radiative channel in the neat perovskite films. When the perovskite films are covered with hole transporting materials, the PL decay rate for all three films becomes significantly reduced. The strong photoluminescence quenching indicates efficient extraction of holes at the perovskite/HTM interfaces.

To evaluate the efficiency of the charge transfer properties from perovskite to HTMs from time‐resolved PL measurements, we used previously proposed protocol.^31^ The calculated charge‐transfer time and efficiency values are presented in **Table** **1**. The charge transfer efficiency values of perovskite solar cell devices comprising new HTMs are comparable to that obtained with commercially available Spiro‐OMeTAD. The superior hole collection was also consistent with photovoltaic measurements of devices, which showed similar power conversion efficiencies between all devices.

**Table 1:** Charge–transfer time (τCT) and efficiency (CTE) calculated for perovskite films employing various HTMs

HTM	τ [ns]	CTE [%]
**V1050**	12.8	81.9
**V1061**	15.9	78.4
Spiro‐OMeTAD	9.6	85.8

### 2.4 Photoelectrical Properties

**Figure 5:**
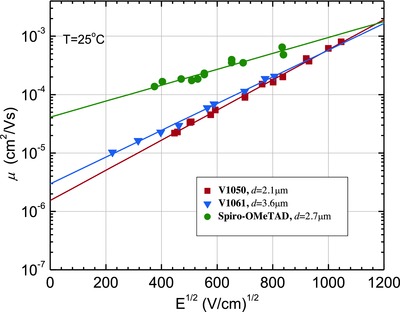
Electric field dependencies of the hole‐drift mobilities (*µ*) in charge transport layers of **V1050**, **V1061**, and Spiro‐OMeTAD, with corresponding linear fits represented by solid lines.

Xerographic time‐of‐flight technique was used to characterize charge transporting properties of the synthesized HTMs. The measured charge mobility values of **V1050** and **V1061** are found to be comparable to the values measured for Spiro‐OMeTAD. Values of charge mobility defining parameters: zero field mobility (µ_0_) and the mobility at the electric field of 6.4 × 10^5^ V cm^−1^ are given in **Table** **2**. The measured hole–drift mobility for **V1061** was 3.0 × 10^−6^ cm^2^ V^−1^ s^−1^, while for **V1050** – 1.5 × 10^−6^ cm^2^ V^−1^ s^−1^ at weak electric fields. Interestingly, the charge mobility values of the new HTMs at strong field strength coming closer to the values measured for Spiro‐OMeTAD (**Figure** **5**, Table 2). Lower hole drift mobility values could be attributed to the presence of the bulky central fluorene fragment, which is linked to the 4,4′‐dimethoxydiphenylamine 3,6‐disubstituted carbazole‐based hole transporting moieties by nonconjugated bonds. Conformational freedom increases disorder of molecules in the films, thus lowering mobility values.

**Table 2:** Thermal and photoelectrical properties of **V1050**, **V1061**, and Spiro‐OMeTAD

HTM	*T*_g_ a) [°C]	*T*_dec_ b) [°C]	*I*_p_ c) [eV]	*µ*_0_ d) [cm^2^ V^−1^ s^−1^]	*µ* e) [cm^2^ V^−1^ s^−1^]
**V1050**	166	400	5.11	1.5 × 10^−6^	1.7 × 10^−4^
**V1061**	146	420	5.10	3.0 × 10^−6^	2.0 × 10^−4^
Spiro‐OMeTAD^26^	126	449	5.00	4.1 × 10^−5^	5.0 × 10^−4^

^a)^ Determined by DSC: scan rate = 10 °C min^−1^, N_2_ atmosphere; second run

^b)^ Thermal decomposition temperature was registered at 5% weight loss

^c)^ Ionization potential was measured by the photoemission in air method from films

^d)^ Mobility value at zero field strength

^e)^ Mobility value at 6.4 × 10^5^ V cm^−1^ field strength.

To determine the highest occupied molecular orbital (HOMO) energy level of **V1050** and **V1061**, solid state ionization potential (*I*_p_) was measured by photoelectron spectroscopy in air method^32^ and results are presented in Table 2; the measurement error is evaluated as 0.03 eV. Compounds **V1050** and **V1061** have almost the same *I*_p_ values, 5.11 and 5.10 eV, respectively (Figures S3 and S4, Supporting Information). The estimated *I*_p_ values are very closed to the value of Spiro‐OMeTAD (5.00 eV) and compatible for application in perovskite solar cell devices to ensure efficient hole transfer at the interface.

### 2.5 Perovskite Solar Cells

Currently one of the best and most reproducible results are obtained with “triple‐cation” composition of perovskite, containing MA^+^, FA^+^, and inorganic Cs^+^ cations.^3^ However, MA^+^ is a volatile cation and can cause degradation of the perovskite absorber film. Thus, during the last years, MA^+^‐free architectures have been extensively studied, where FA^+^ cation is combined with Cs^+^, which provides higher stability.^33^, ^34^, ^35^, ^36^ SnO_2_ was used as an electron transporting layer, as it can be deposited at low temperatures (annealing temperature–180 °C), and thus is suitable material for the application in tandem solar cells.^37^

**Figure 6:**
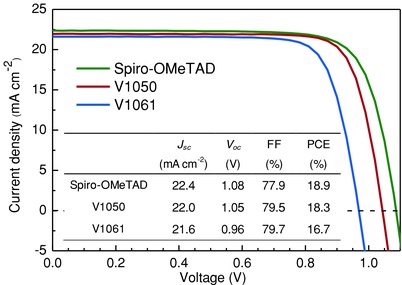
Best performing *J*–*V* characteristics (reverse scan) of the best devices employing **V1050**, **V1061**, and Spiro‐OMeTAD.

The nonspiro, fluorene‐based compounds **V1050** and **V1061** were tested in perovskite solar cells employing planar FTO/SnO_2_/FA_0.83_Cs_0.17_Pb(I_0.8_Br_0.2_)_3_/HTM/Au architecture. **Figure** **6** shows current–voltage characteristics of the best devices employing **V1050**, **V1061**, and Spiro‐OMeTAD in planar PSC. The optimized champion device efficiency for **V1050**, **V1061**, Spiro‐OMeTAD, and corresponding photovoltaic performance parameters are shown in Figure 6. The **V1050** HTM‐based device shows the device efficiency of 18.3% (*J*_sc_ = 22.0 mA cm^−2^, *V*_OC_ = 1.05 V, and *FF* = 79.5%). This is comparable to the state‐of‐the‐art material Spiro‐OMeTAD on a like‐to‐like comparison (PCE = 18.9%, *J*_sc_ = 22.4 mA cm^−2^, *V*_OC_ = 1.08 V, and *FF* = 77.9%). A bit lower PCE of 16.7% (Figure 6) was recorded with *J*_sc_ = 21.6 mA cm^−2^, *V*_OC_ = 0.96 V, and *FF* = 79.7% in case of **V1061**. Efficiencies at stabilized power output (SPO) of tested compounds are shown in Figure S5 in the Supporting Information and are 16.1%, 15.0%, and 17.3%, respectively. External quantum efficiency (EQE) spectra were recorded for typical device for each HTMs in Figure S6 (Supporting Information). The same trend was observed from the measured *J*–*V* curves. The statistical distribution of the PSC parameters employing investigated HTMs is shown in Figure S7 in the Supporting Information and is indicating high reproducibility of PSC devices. Furthermore, Figure S8 (Supporting Information) shows *J*–*V* curves from both forward‐bias to short‐circuit and short‐circuit to forward‐bias current–voltage sweeps of typical device from both sweeps. While hysteresis is quite pronounced, it is comparable between the mentioning HTMs, thus reverse scans can be used as representatives for the HTMs performance analysis.

**Figure 7:**
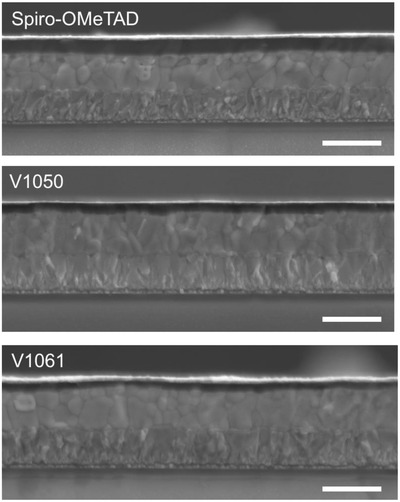
Cross‐sectional SEM microscopy image of the devices with Spiro‐OMeTAD, **V1050**, and **V1061** as a HTM. Scale bar equals to 1000 nm.

From the SEM cross‐section images, it can be seen, that new fluorene‐based compounds form a uniform morphology on top of perovskite layer as similar as Spiro‐OMeTAD (**Figure** **7**).

To give good performance, HTM should effectively block the electrons and transport holes from perovskite to the Au electrode. Thus, it is important for the HTMs to have excellent film forming ability. As from the SEM cross‐section images no significant difference could be observed, contact angle measurement of the HTM droplets on the perovskite film was performed (Figure S9, Supporting Information). From the results it can be seen, that **V1050** solution has the lowest contact angle of 9°, which could potentially lead to a better film forming ability of **V1050**.

**Figure 8:**
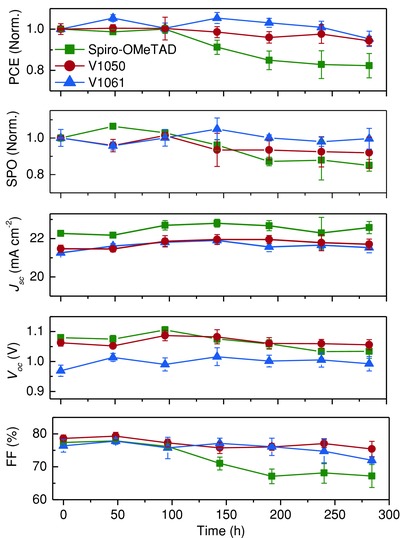
Transition of the PV performance parameters (PCE, SPO, *J*_sc_, *V*_oc_, and *FF*) of the PSCs with **V1050**, **V1061**, and Spiro‐OMeTAD.

Additionally, we also studied the stability of fabricated champion devices without any encapsulation at uncontrollable humidity conditions (relative humidity ≈ 60%, temperature 22 °C, in dark condition) for our newly developed **V1050**, **V1061** and standard Spiro‐OMeTAD HTMs. It can be seen (**Figure** **8**) that the PCE values diminished down ≈20% after 330 h for Spiro‐OMeTAD based devices. However, the devices based on **V1050** and **V1061** are seen to be more stable with observed efficiency reduced only ≈6% at the same conditions. The device stability test results revealed that the improved stability of **V1050** and **V1061**‐based cells compared to Spiro‐OMeTAD‐based device could be attributed to the uniform HTM capping layer on the top of the perovskite layer, preventing the moisture penetration into the perovskite layer, which is in agreement with the result from HTM contact angle measurement on top of perovskite film. The overall good performance of PCE and stability of **V1050** over Spiro‐OMeTAD provide a promising alternative replacement for high performance PSCs.

## 3 Conclusions

In conclusion, a new promising nonspiro fluorene‐based hole transport materials **V1050** and **V1061** were synthesized and characterized. The synthesis of these HTMs consists of three steps starting from the commercially available materials. Solar cells using **V1050** exhibit PCEs of 18.3% which is comparable to the performance of PSC comprising Spiro‐OMeTAD (18.9%) as HTM. Compared with Spiro‐OMeTAD, new HTM additionally shows several significant advantages: it has much facile synthesis, has high glass transition temperature (166 °C) and does not form the crystalline state. Moreover, this new HTM also exhibits better environmental stability compared to Spiro‐OMeTAD. We believe that the **V1050** can be a useful alternative HTM to Spiro‐OMeTAD for perovskite solar cells, thus bringing PSCs closer to commercial production.

## Conflict of Interest

The authors declare no conflict of interest.

## Supporting information

Supplementary
