# Supplementary material for: Nonspiro, Fluorene‐Based, Amorphous Hole Transporting Materials for Efficient and Stable Perovskite Solar Cells
Source: Adv Sci (Weinh). 2018 Jan 31;5(4):1700811. doi: 10.1002/advs.201700811 (PMC5908358; doi:10.1002/advs.201700811)
Supplement: Supplementary file 1 — Supplementary [file ADVS-5-1700811-s001.pdf]

Copyright WILEY-VCH Verlag GmbH & Co. KGaA, 69469 Weinheim, Germany, 2018.

ADVANCED SCIENCE
Open Access

Supporting Information

for *Adv. Sci.,* DOI: 10.1002/advs.201700811

Nonspiro, Fluorene-Based, Amorphous Hole Transporting Materials for Efficient and Stable Perovskite Solar Cells

Šaruūnė Daškevičiūtė, Nobuya Sakai, Marius Franckevičius, Marytė Daškevičienė, Artiom Magomedov, Vygintas Jankauskas, Henry J. Snaith, and Vytautas Getautis**

Supporting information

Non-spiro, fluorene-based, amorphous hole transporting material for efficient and stable perovskite solar cells

Šarūnė Daškevičiūtė,†[a] Nobuya Sakai,†[b] Marius Franckevičius,[c] Marytė Daškevičienė,[a] Artiom Magomedov,[a] Vygintas Jankauskas,[d] Henry J. Snaith,*[b] Vytautas Getautis*[a]

[a] Department of Organic Chemistry, Kaunas University of Technology, Radvilenu pl. 19, Kaunas, 50254, Lithuania, E-mail: vytautas.getautis@ktu.lt

[b] Department of Physics Clarendon Laboratory, University of Oxford, Parks Road, Oxford, OX1 3PU, UK, E-mail: henry.snaith@physics.ox.ac.uk

[c] Center for Physical Sciences and Technology, Saulėtekio Ave. 3, Vilnius, LT-10257, Lithuania

[d] Institute of Chemical Physics Vilnius University Saulėtekio al. 3, Vilnius, 10257, Lithuania

† These authors contributed equally to this work

Contents

General methods and materials 2

Synthesis 2

^{1}H NMR and ^{13}C NMR spectra of **V1050** and **V1061** 6

Hole Drift Mobility Measurements 10

Thermal Properties 10

Ionization Potential Measurements 12

Device fabrication 13

Absorption and photoluminescence lifetime measurements 14

Device characterization 14

References 18

General methods and materials

Chemicals were purchased from Sigma-Aldrich and TCI Europe and used as received without further purification. The ^{1}H and ^{13}C NMR spectra were taken on Bruker Avance III (400 MHz) spectrometer at RT. All the data are given as chemical shifts in δ (ppm). The course of the reactions products was monitored by TLC on ALUGRAM SIL G/UV254 plates and developed with UV light. Silica gel (grade 9385, 230–400 mesh, 60 Å, Aldrich) was used for column chromatography. Elemental analysis was performed with an Exeter Analytical CE-440 elemental analyser, Model 440 C/H/N/. Differential scanning calorimetry (DSC) was performed on a Q10 calorimeter (TA Instruments) at a scan rate of 10 K min^{-1} in the nitrogen atmosphere. The glass transition temperatures for the investigated compounds were determined during the second heating scan. Electrothermal MEL-TEMP capillary melting point apparatus was used for determination of melting points. UV/vis spectra were recorded on Shimadzu UV-3600 spectrometer. A scanning electron microscope (SEM; Hitachi, S-4300) was used to acquire cross section SEM image of the perovskite solar cells. Contact angle measurement was performed by Kruss, Drop Shape Analysis System DSA25.

Synthesis

2,7-Bis(bromomethyl)-9,9-diethyl-9*H*-fluorene (1)

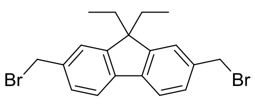

A mixture of 9,9-diethyl-9*H*-fluorene (1.11 g, 5 mmol), paraformaldehyde (0.33 g, 11 mmol), and 33% HBr solution in acetic acid (10 ml) was heated at 60-70 °C for 20 h. Upon cooling, the precipitates were filtered off and tree times washed with water and dried in vacuum, affording 1.47 g of pale white solid (72.0%). The product was recrystallized from toluene/*n*-hexane 1:1 gave as white crystals. Mp 148-150°C.
The NMR spectra were identical to the corresponding spectra of the product referred in [1];
Anal. calcd for C19H20Br2: C, 55.91; H, 4.94; found: C, 55.68; H, 4.81.

2,7-Bis(3,6-dibromo-9*H*-carbazol-9-methyl)-9,9-diethyl-9*H*-fluorene (2)

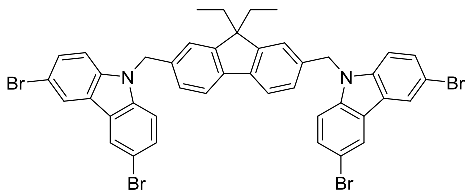

A mixture of compound **1** (0.82 g, 2 mmol) and 3,6-dibromo-9*H*-carbazole (1,30 g, 4 mmol) was dissolved in 15 ml of tetrahydrofurane and 0.68 g (12 mmol) of 85% powdered potassium hydroxide was added in small portions during 2-3 minutes. The obtained mixture was stirred at room temperature for 6 h. The part of solvent was removed in vacuum. Then obtained crystals of product **2** were filtered off and washed with water until it was neutral and three times with ethanol. The product was recrystallized from ethanol/tetrahydrofurane 2:1 gave as white crystals (1.82 g, 83.1%), Mp 293-295°C.

^{1}H NMR (400 MHz, DMSO-d_6) δ: 8.49 (s, 4H), 7.72-7.48 (m, 10H), 7.35 (s, 2H), 6.94 (d, *J* = 7.8 Hz, 2H), 5.70 (s, 4H), 1.83 (q, *J* = 7.2 Hz, 2H), 1.81 (d, *J* = 7.9 Hz, 4H), 0.10 (t, *J* = 7.2 Hz, 6H) ppm.
^{13}C NMR (101 MHz, DMSO) δ: 150.09, 139.95, 139.49, 136.31, 129.08, 125.65, 123.73, 123.37, 122.21, 120.21,112.21, 111.79, 55.43, 46.59, 31.77, 8.47 ppm.
Anal. calcd for C43H32Br4N2: C, 57.64; H, 3.60; N, 3.13; found: C, 57.18; H, 3.31; N, 2.98.

2,4,7-Tris(bromomethyl)-9,9-dimethyl-9*H*-fluorene (3)

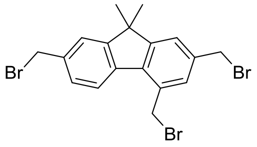

A mixture of 9,9-dimethyl-9*H*-fluorene (1) (2.50 g, 12.9 mmol), paraformaldehyde (3.86 g, 129 mmol), and 33% HBr solution in acetic acid (25 mL) was heated at 85-90 ºC for 45 h. Upon cooling, the precipitate was collected by filtration, carefully washed with water and then with ether. The brown crude product was purified by column chromatography on silica gel, using 1:24 v/v acetone/n-hexane as an eluent, affording 3.90 g of white solid (64.1%). The product was recrystallized from *n*-hexane gave as white crystals. Mp 164-165°C.
^{1}H NMR (400 MHz, DMSO-d_6) δ: 7.95 (d, *J* = 8.0 1H), 7.79-7.64 (m, 2H), 7.61-7.48 (m, 2H), 5.09 (s, 2H), 4.82 (s, 2H), 4.78 (s, 2H), 1.48, 1.44 (two s, 6H) ppm.
^{13}C NMR (101 MHz, DMSO) δ: 152.21, 151.30, 139.87, 139.11, 137.89, 137.79, 132.88, 131,12, 129.18, 124.63, 124.58, 124.13, 55.74, 34.93, 34.72, 33.86, 32.27 ppm.

Anal. calcd for C18H17Br3: C, 45.70; H, 3.62; found: C, 45.58; H, 3.51.

2,4,7-Tris(3,6-dibromo-9*H*-carbazol-9-methyl)-9,9-dimethyl-9*H*-fluorene (4)

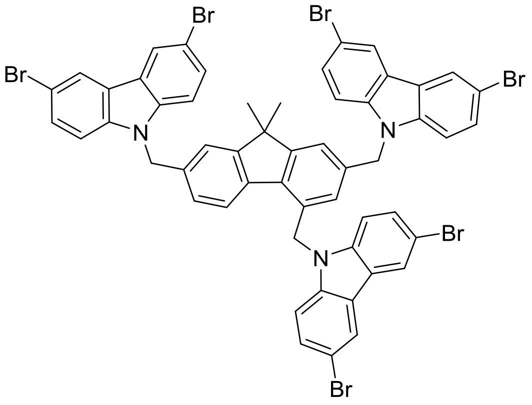

A mixture of compound **3** (1.42 g, 3 mmol) and 3,6-dibromo-9*H*-carbazole (2.93 g, 9 mmol) was dissolved in 30 ml of tetrahydrofurane and 1,52 g (27 mmol) of 85% powdered potassium hydroxide was added in small portions during 2-3 minutes. The obtained mixture was stirred at room temperature for 6 h. The resulting solid was dissolved in 80 mL tetrahydrofurane and washed with water (3 × 50 mL). Then the organic layer was separated and dried over Na_2SO_4. The crude product was purified by column chromatography on silica gel using 4:21 v/v tetrahydrofurane/*n*-hexane as an eluent gave the product **4** as white crystals (2.83 g, 78.3%), Mp 204-205°C.

^{1}H NMR (400 MHz, DMSO-d_6) δ: 8.52 (s, 2H), 8.19 (d, *J* = 18.2 Hz, 4H), 7.81 (d, *J* = 8.0 Hz 1H), 7.71 (d, *J* = 8.0 Hz, 2H), 7.66-7.59 (m, 3H), 7.47 (s, 1H), 7.24-7.16 (m, 4H), 7.00 (dd, *J* = 19.4 Hz, *J* = 8.8 Hz, 4H), 5.78 (s, 4H), 5.32 (s, 2H), 4.84 (s, 1H), 1.42 (s, 6H) ppm.
Anal. calcd for C54H35Br6N3: C, 53.81; H, 2.93; N, 3.49; found: C, 53.48; H, 2.61; N, 3.18.

2,7-Bis[3,6-di(4,4`-dimethoxy)diphenylamino-9*H*-carbazol-9-methyl]-9,9-diethyl-9*H*-fluorene (V1061)

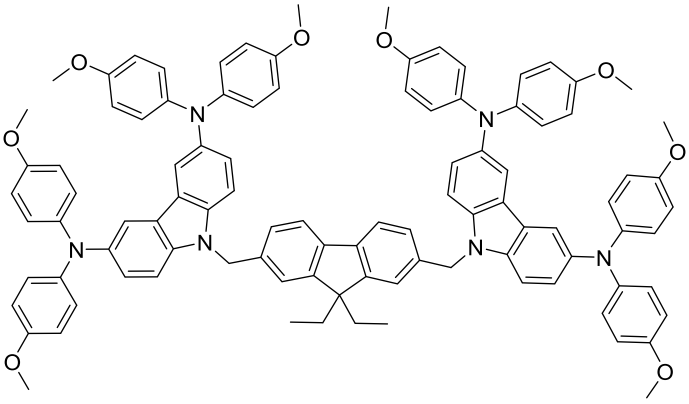

A solution of compound **2** (1.34 g, 1.5 mmol), 4,4`-dimethoxydiphenylamine (2.06 g, 9 mmol) in anhydrous toluene (17 mL) was purged with argon for 30 minutes. Afterwards, palladium(II) acetate (6.7 mg, 0.03 mmol), tri-*tert*-butylphosphonium tetrafluoroborate (11.7 mg, 0.04 mmol) and sodium *tert*-butoxide (0.86 g, 9 mmol) were added and the solution was refluxed under argon atmosphere for 24 hours. After cooling to room temperature, reaction mixture was filtered through Celite, 50 mL of distilled water were added and extraction was done with ethyl acetate and distilled water. The organic layer was dried over anhydrous Na_2SO_4, filtered and solvent evaporated. The crude product was purified by column chromatography using 1:4 v/v acetone/*n*-hexane as an eluent. The obtained product was precipitated from tetrahydrofurane into 15-fold excess of hexane. The precipitate was filtered off and washed with hexane to collect **V1061** as a pale yellow – green solid. (1.70 g, 77.6%).

^{1}H NMR (400 MHz, DMSO-d_6) δ: 7.65 (s, 4H), 7.57 (d, J = 7.8 Hz, 2H), 7.50 (d, J = 8.8 Hz, 4H), 7.34 (s, 2H), 7.04 (d, J = 8.4, 6H), 6.90 – 6.69 (m, 32H), 5.58 (s, 4H), 3.65 (s, 24H), 1.81 (m, 4H), 0.12 (t, J = 7.3 Hz, 6H) ppm.
^{13}C NMR (101 MHz, DMSO) δ: 154.63, 150.38, 142.50, 140.69, 140.10, 137.88, 137.07, 126.09, 125.79, 124.76, 124.15, 123.36, 122.56, 120.40, 117.27, 115.06, 111.18, 55.60, 46.90, 31.96, 26.81, 8.79 ppm.
Anal. calcd for C99H88N6O8: C, 79.81; H, 5.95; N, 5.64; found: C, 79.48; H, 5.71; N, 5.68.

2,4,7-Tris[3,6-di(4,4`-dimethoxy)diphenylamino-9*H*-carbazol-9-methyl]-9,9-dimethyl-9*H*-fluorene (V1050)

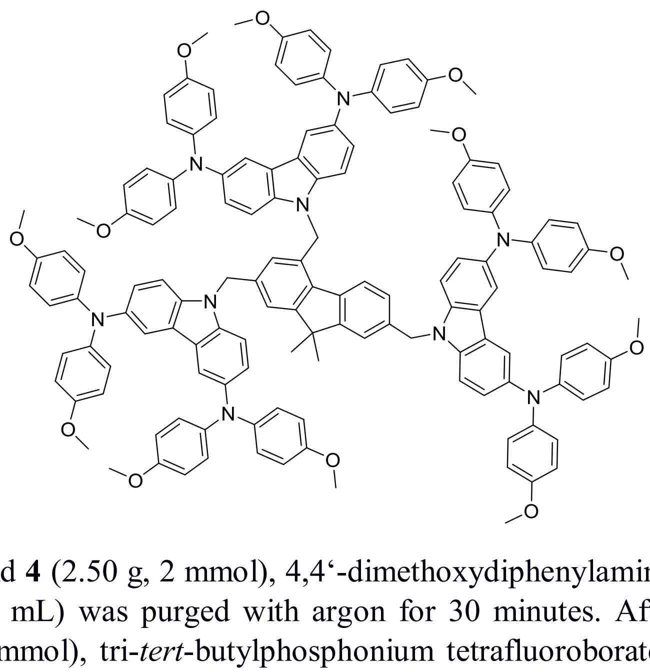

A solution of compound **4** (2.50 g, 2 mmol), 4,4‘-dimethoxydiphenylamine (4.19 g, 18 mmol) in anhydrous toluene (35 mL) was purged with argon for 30 minutes. Afterwards, palladium(II) acetate (9.3 mg, 0.04 mmol), tri-*tert*-butylphosphonium tetrafluoroborate (6.5 mg, 0.02 mmol) and sodium *tert*-butoxide (1.8 g, 18 mmol) were added and the solution was refluxed under argon atmosphere for 52 hours. After cooling to room temperature, reaction mixture was filtered

through Celite, 50 mL of distilled water were added and extraction was done with ethyl acetate and distilled water. The organic layer was dried over anhydrous Na_2SO_4, filtered and solvent evaporated. The crude product was purified by column chromatography using 6:3.5:15.5 v/v acetone/tetrahydrofurane/*n*-hexane as an eluent. The obtained product was precipitated from toluene into 15-fold excess of hexane. The precipitate was filtered off and washed with hexane to collect **V1050** as a pale yellow – green solid (3.03 g, 71.8%).

^{1}H NMR (400 MHz, DMSO-d_6) δ 7.85 (d, J = 8.2 Hz, 1H), 7.73 – 7.44 (m, 9H), 7.33 (s, 1H), 7.26 – 6.56 (m, 60H), 6.34 (s, 1H), 5.88 (s, 2H), 5.61 (s, 2H), 5.14 (s, 2H), 3.77 – 3.50 (m, 36H), 1.28 (s, 6H).
^{13}C NMR (101 MHz, DMSO) δ 154.64, 154.60, 154.58, 142.50, 142.45, 140.78, 140.54, 137.97, 137.82, 137.68, 135.04, 124.84, 124.20, 124.14, 123.34, 123.22, 117.08, 115.07, 115.02, 114.97, 55.59, 55.55, 55.53, 46.36, 40.67, 40.62, 40.47, 40.41, 40.26, 40.21, 40.05, 40.00, 39.79, 39.58, 39.37, 27.41.
Anal. calcd for C138H1194N9O12: C, 79.10; H, 5.72; N, 6.02; found: C, 78.88; H, 5.61; N, 5.88.

^{1}H NMR and ^{13}C NMR spectra of V1061 and V1050

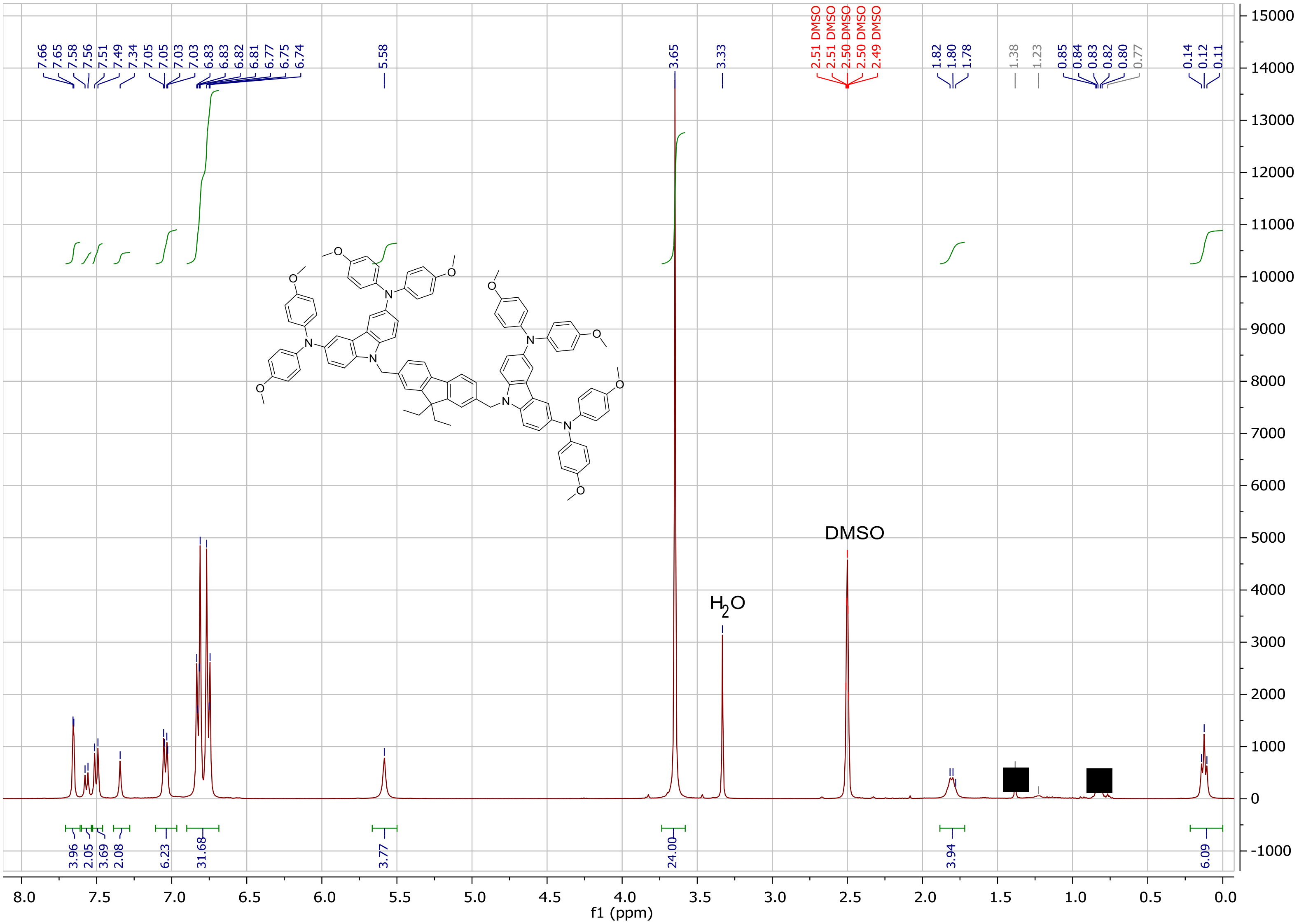

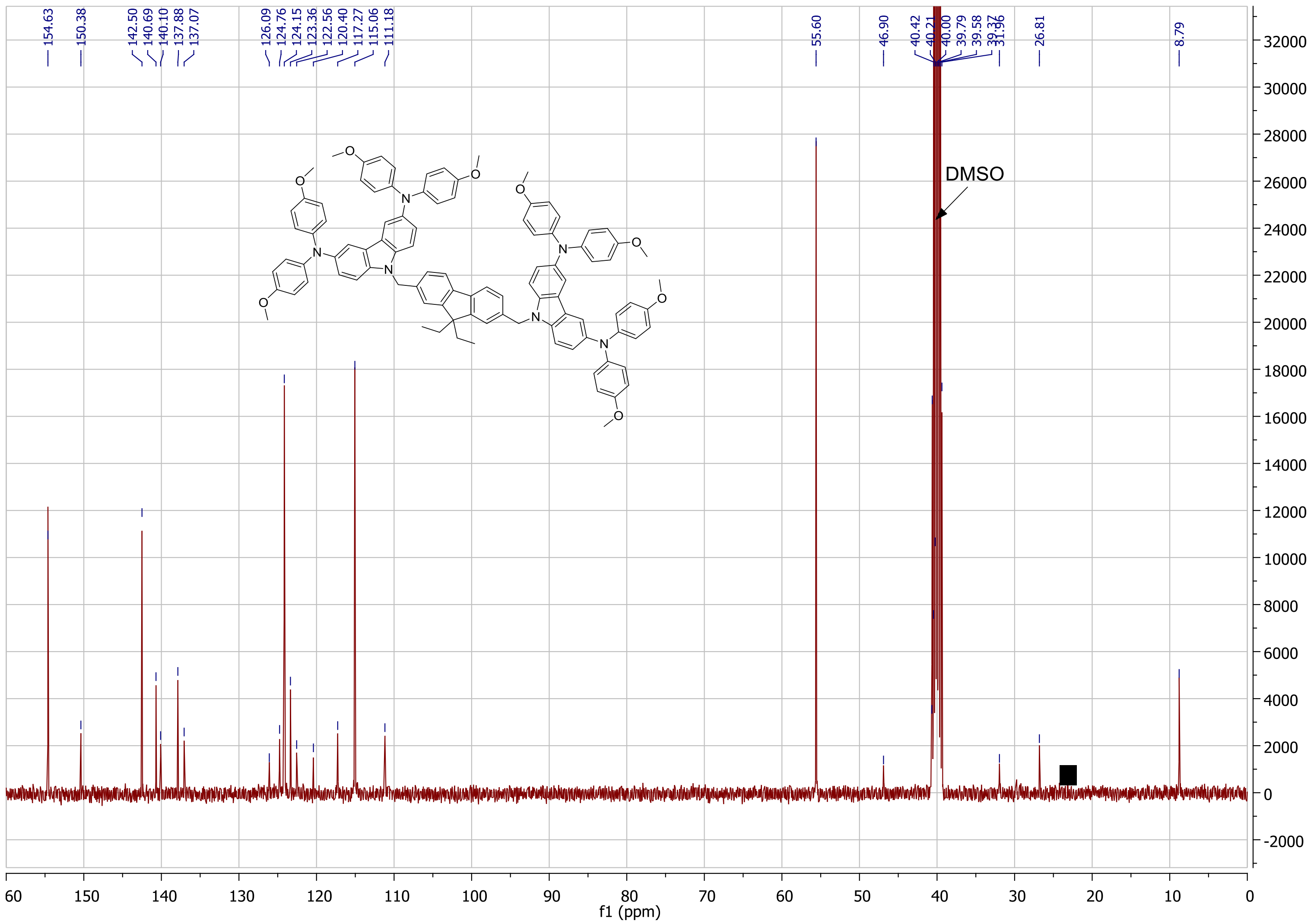
154.63
150.38
142.50
140.69
140.10
137.88
137.07
126.09
124.76
124.15
123.36
122.56
120.40
117.27
115.06
111.18
55.60
46.90
40.42
40.21
40.00
39.79
39.58
39.37
31.96
26.81
8.79
DMSO
f1 (ppm)

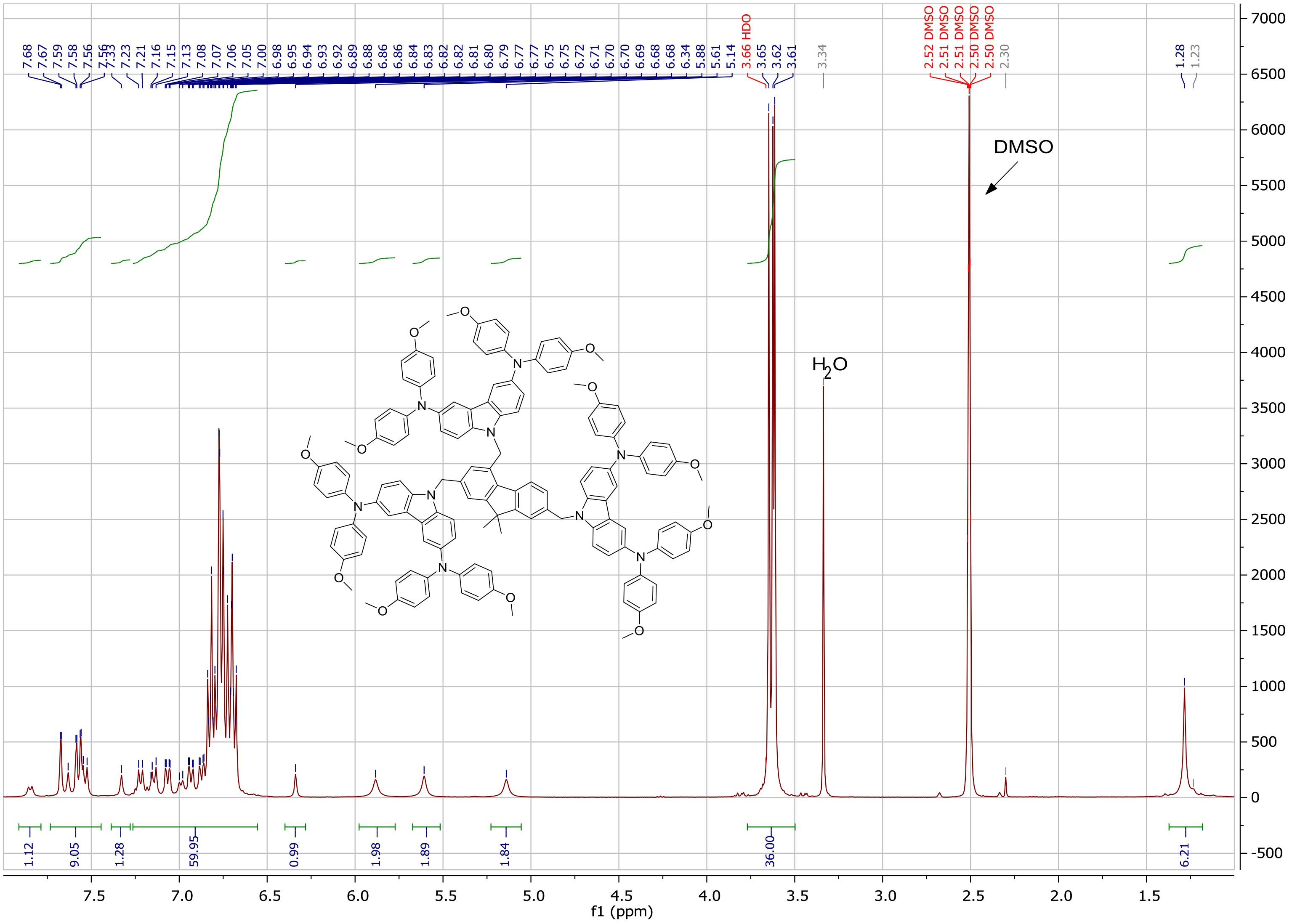
7.68
7.67
7.59
7.58
7.56
7.56
7.33
7.23
7.21
7.16
7.15
7.13
7.08
7.07
7.06
7.05
7.00
6.98
6.95
6.94
6.93
6.92
6.89
6.88
6.86
6.86
6.84
6.83
6.82
6.82
6.81
6.80
6.79
6.77
6.77
6.75
6.75
6.72
6.71
6.70
6.70
6.69
6.68
6.68
6.34
5.88
5.61
5.14
3.66 HDO
3.65
3.62
3.61
3.34
2.52 DMSO
2.51 DMSO
2.51 DMSO
2.50 DMSO
2.50 DMSO
2.30
1.28
1.23
DMSO
H_2O
1.12
9.05
1.28
59.95
0.99
1.98
1.89
1.84
36.00
6.21
f1 (ppm)

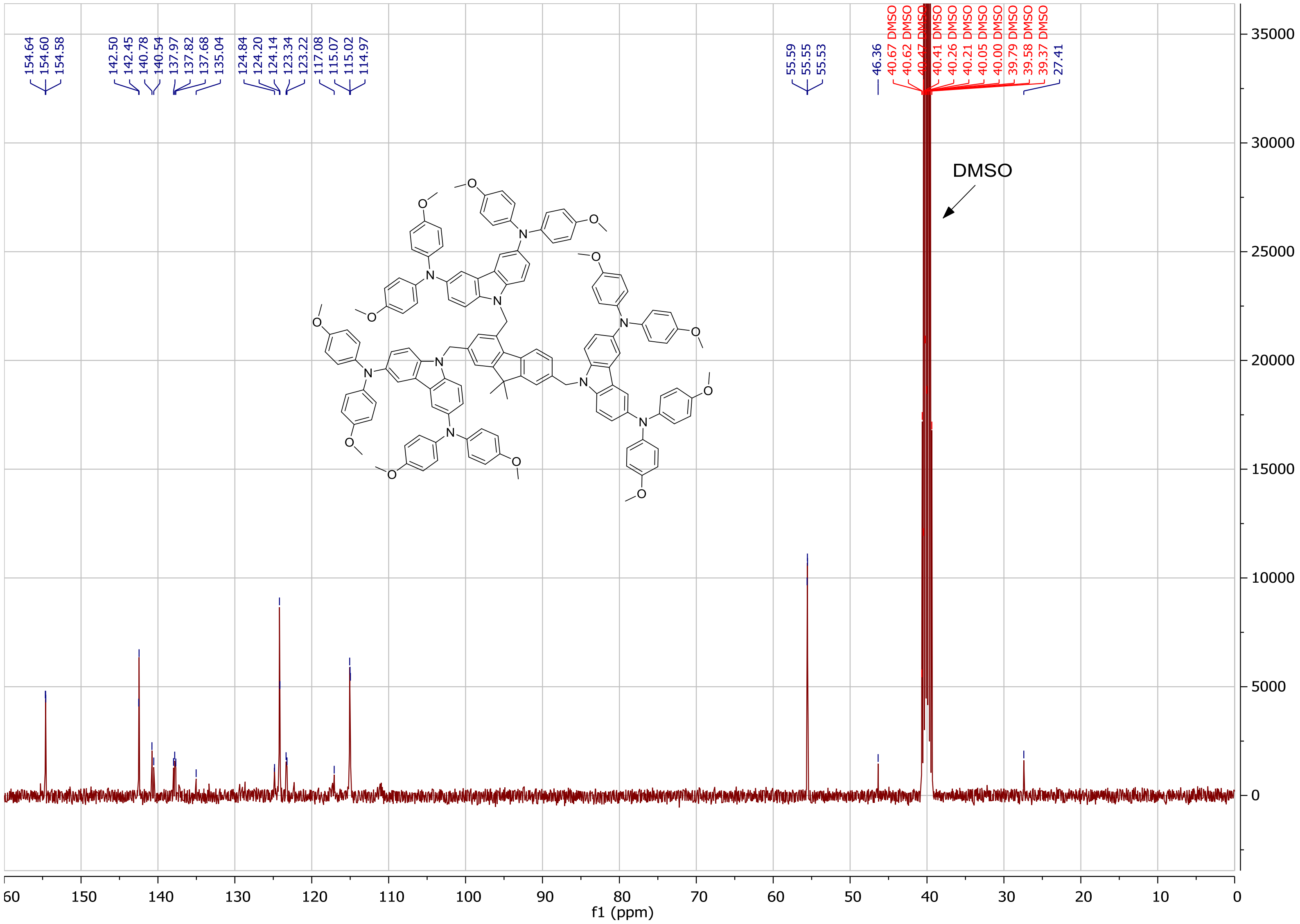

Hole drift mobility measurements

The samples for the hole mobility measurements were prepared by spin-coating the solution of the HTMs on the polyester films with conductive Al layer. The layer thickness was in the range of 2-4 µm. The hole drift mobility was measured by xerographic time of flight technique (XTOF) [2–4]. Electric field was created by positive corona charging. The charge carriers were generated at the layer surface by illumination with pulses of nitrogen laser (pulse duration was 2 ns, wavelength 337 nm). The layer surface potential decrease as a result of pulse illumination

was up to 1-5 % of initial potential before illumination. The capacitance probe that was connected to the wide frequency band electrometer measured the speed of the surface potential decrease dU/dt. The transit time t_t was determined by the kink on the curve of the dU/dt transient in double logarithmic scale. The drift mobility was calculated by the formula $\mu = d^2/U_0 t_t$, where d is the layer thickness, U_0 – the surface potential at the moment of illumination.

Thermal properties

Differential scanning calorimetry (DSC) was performed on a Q10 calorimeter (TA Instruments) at a scan rate of 10 K min^{-1} in the nitrogen atmosphere. The glass transition temperatures for the investigated compounds were determined during the second heating scan.

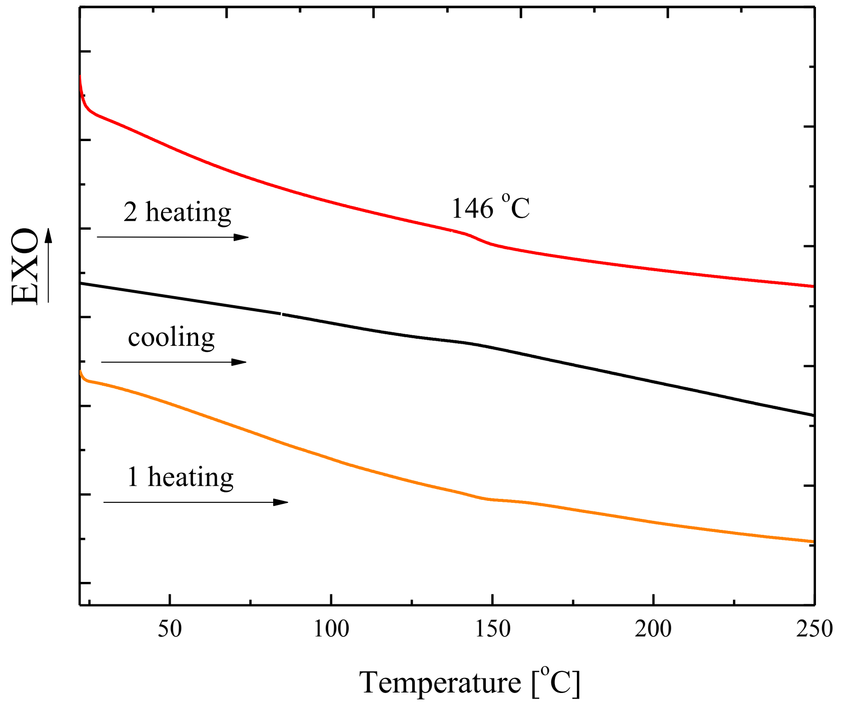

Figure S1. Photoemission in air spectra of the ***V1061***

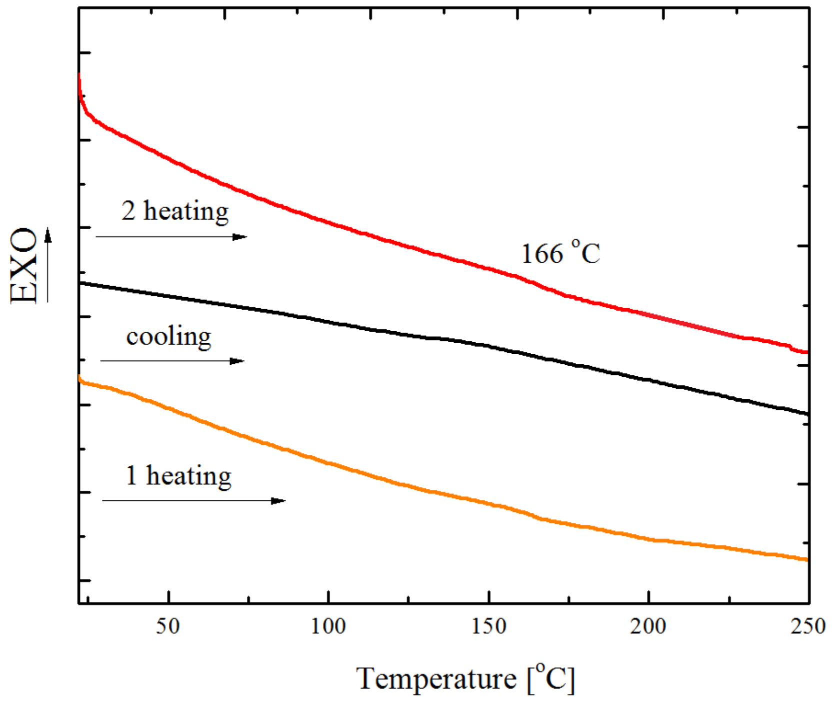

*Figure S2. Photoemission in air spectra of the **V1050***

Ionization potential measurements

The solid state ionization potential (I_p) of the layers of the synthesized compounds was measured by the electron photoemission in air method [5–7]. The samples for the ionization potential measurement were prepared by dissolving materials in $CHCl_3$ and were coated on Al plates pre-coated with ~0.5 µm thick methylmethacrylate and methacrylic acid copolymer adhesive layer. The thickness of the transporting material layer was 0.5-1 µm. Usually photoemission experiments are carried out in vacuum and high vacuum is one of the main requirements for these measurements. If vacuum is not high enough the sample surface oxidation and gas adsorption are influencing the measurement results. In our case, however, the organic materials investigated are stable enough to oxygen and the measurements may be carried out in the air. The samples were illuminated with monochromatic light from the quartz monochromator with deuterium lamp. The power of the incident light beam was (2-5)$\cdot 10^{-8}$ W. The negative voltage of –300 V was supplied to the sample substrate. The counter-electrode with the 4.5×15 mm^2 slit for

illumination was placed at 8 mm distance from the sample surface. The counter-electrode was connected to the input of the BK2-16 type electrometer, working in the open input regime, for the photocurrent measurement. The $10^{-15} – 10^{-12}$ A strong photocurrent was flowing in the circuit under illumination. The photocurrent *I* is strongly dependent on the incident light photon energy hν. The $I^{0.5} = f(h\nu)$ dependence was plotted. Usually the dependence of the photocurrent on incident light quanta energy is well described by linear relationship between $I^{0.5}$ and *hν* near the threshold [6, 7]. The linear part of this dependence was extrapolated to the hν axis and I_p value was determined as the photon energy at the interception point.

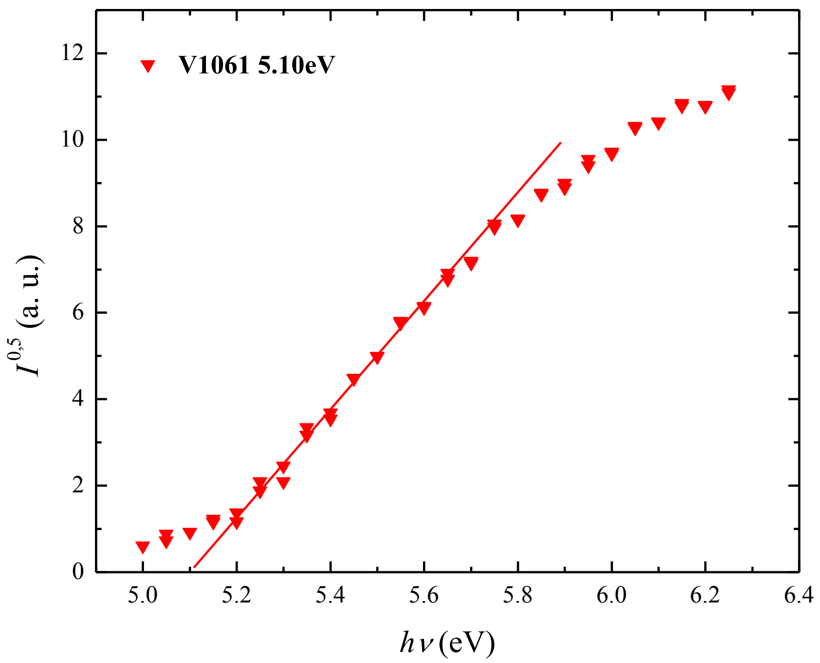

*Figure S4. Photoemission in air spectra of the **V1061***

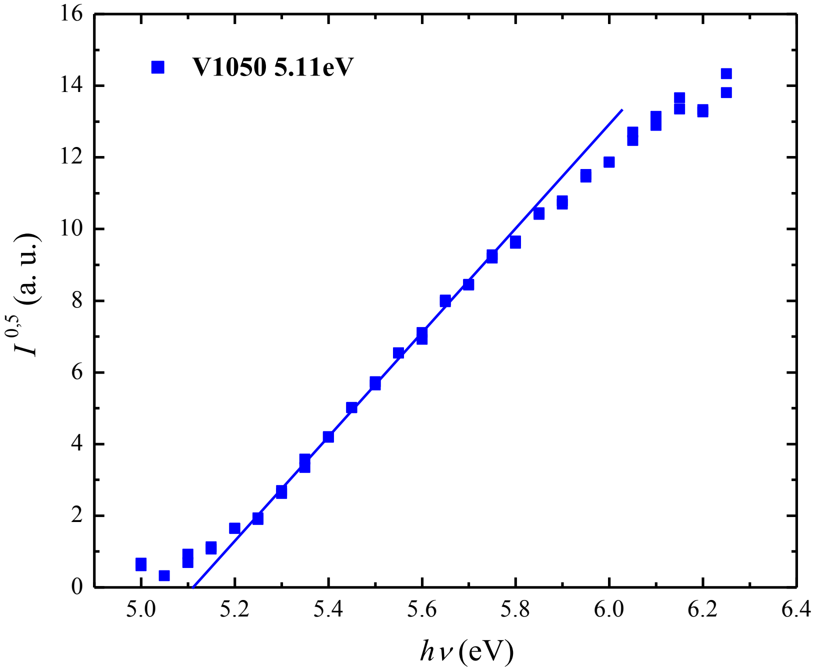

*Figure S4. Photoemission in air spectra of the **V1051***

Absorption and photoluminescence lifetime measurements

Optical absorption spectra of perovskite films were recorded with a UV-Vis spectrophotometer (Jasco) in transmission mode in 550-850 nm spectral range. Fluorescence decay kinetics were measured using the Edinburgh Instruments time-correlated single photon counting fluorescence spectrometer F900. The picosecond pulsed diode laser EPL-470 emitting 72 ps pulses at 470 nm with the repetition rate of 50 MHz (100 ns) was used for the sample excitation. The time resolution of the setup was about several hundreds of picoseconds by applying apparatus function deconvolution.

Device fabrication

FTO-coated glass sheets (TEC 7, 7Ω/sheet, Pilkington) were etched with zinc powder and 2M HCl to obtain the required electrode pattern. The sheets were then washed with 2% Hellmanex in water, deionized water, acetone, ethanol and iso-propanol. The last traces of organic residues were removed by oxygen plasma cleaning for 10 min. An SnO_2 compact layer was fabricated

from SnO_2 precursor, which were fabricated via spin coating and chemical bath deposition. For spin coating SnO_2, 17.5 mg of $SnCl_4 \cdot 5H_2O$ (Sigma-Aldrich, 98%) was dissolved in 1 ml of iso-propanol. After 30 minutes of stirring at room temperature, a fully dissolved, clear solution was obtained. This solution was then filtered with 0.2 mm pore size polytetrafluoroethylene filter. The $SnCl_4$ solution was spun on FTO substrate at 3000 rpm for 30 s (acceleration: 200 rpm), and annealed at 180 ºC for 60 min to form a thin layer of SnO_2. For chemical bath deposition, 108 mg of $SnCl_2 \cdot 2(H_2O)$ (Sigma-Aldrich, 98%), 500 mg of Urea (Sigma-Aldrich, 98%), 500 mL of HCl (37% solution in water, Fisher Scientific) and 10 mL of 3-Mercaptopropionic acid (Sigma-Aldrich, 99%) were dissolved in 40 mL of H_2O. After a fully dissolved the solution, the spin coated SnO_2 substrates were immersed in the $SnCl_2$ solution at 70 ºC for 180 min. The substrates were immediately rinsed with a deionized water bath and then sonicated for 120 s in the deionized water bath. The substrates were then dried with a nitrogen gun, and annealed at 180 ºC for 60 min.

To obtain a $FA_{0.83}Cs_{0.17}Pb(I_{0.8}Br_{0.2})_3$ precursor solutions, FAI (formamidinium iodided), CsI, $PbBr_2$ and PbI_2 were dissolved in a mixed solvent of anhydrous N,N-dimethylformamide (DMF; Aldrich) and anhydrous dimethyl sulfoxide (DMSO; Aldrich) in 4:1 volume ratio to obtain a stoichiometric solution with desired composition and a molar concentration of 1.4 M.

The perovskite precursor solution was coated onto the SnO_2 substrate by a consecutive two-step spin-coating process at 1000 rpm for 10 sec and 6000 rpm for 25 sec under low humidity (15-20% at 20°C) condition with a dry compressed air parch in dry box. 200 μL of Anisole and chlorobenzene in 9:1 volume ratio was dropped onto the substrate on the onto the pre-crystallized perovskite film for 8–10 seconds with second step spin program at 5000 rpm. The perovskite films were drying at 80 °C for 5 min and then 100 °C for 60 min in the box oven without controlling humidity. 0.019 mmol **V1050**, 0.027 mmol **V1061**, and 0.070 mmol 2,2',7,7'-Tetrakis[N,N-di(4-methoxyphenyl)amino]-9,9'-spirobifluorene (Spiro-OMeTAD, Luminescence Technology Corp.) were solved in 1 ml of chlorobenzene and stirred at 80 °C for 60 min. As the additives, 700 mol% of 4-tertbutylpyridine, 71 mol% of Li-TFSI and 17 mol% Co[t-BuPyPz]$_3$[TFSI]$_3$ were added into the hole transporting material solution. Both of Li-TFSI and Co[t-BuPyPz]$_3$[TFSI]$_3$ were prepared the stock solution for 500 mg and 50 mg in 1 ml acetonitrile, respectively. The HTM solutions were coated on the perovskite with spin-coating at

2500 rpm for 40 sec. 80 nm Au metal contact layer was deposited as the counter electrode on the HTM layer by thermal evaporation.

Device characterization

The J-V curves of Solar cells were obtained using Keithley 2400 Series source meter and Newport 67005 light source, under standard illumination (100 mW·cm^{-2} , AM 1.5 G), under ambient conditions. All J-V measurements were done in reverse scan (1.2 V -> 0 V -> -0.25 V), the step voltage was fixed at 20 mV, a delay time between scans at each voltage step was set to 1000 ms. EQE spectra were evaluated a function of wavelength with a xenon lamp, monochromator (Princeton Instruments, Acton SP2150) and Lock-in amplifiers (Stanford Research Systems, RS830) under short-circuit condition.

Device parameters

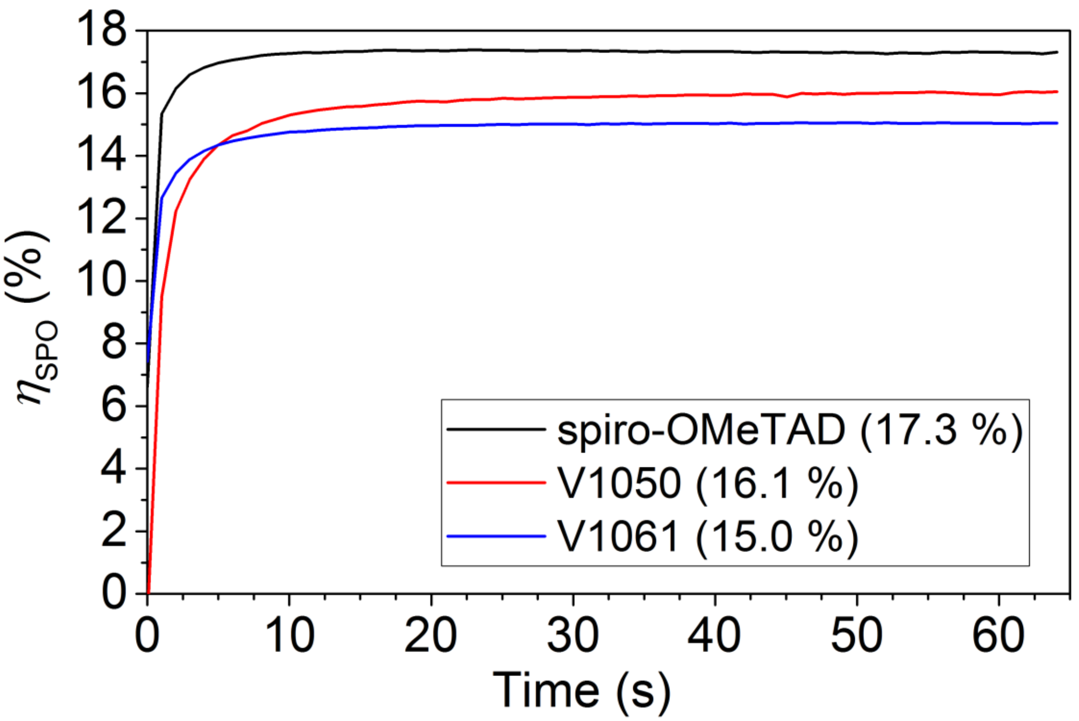

Figure S5. Efficiencies at stabilized power output of tested compounds

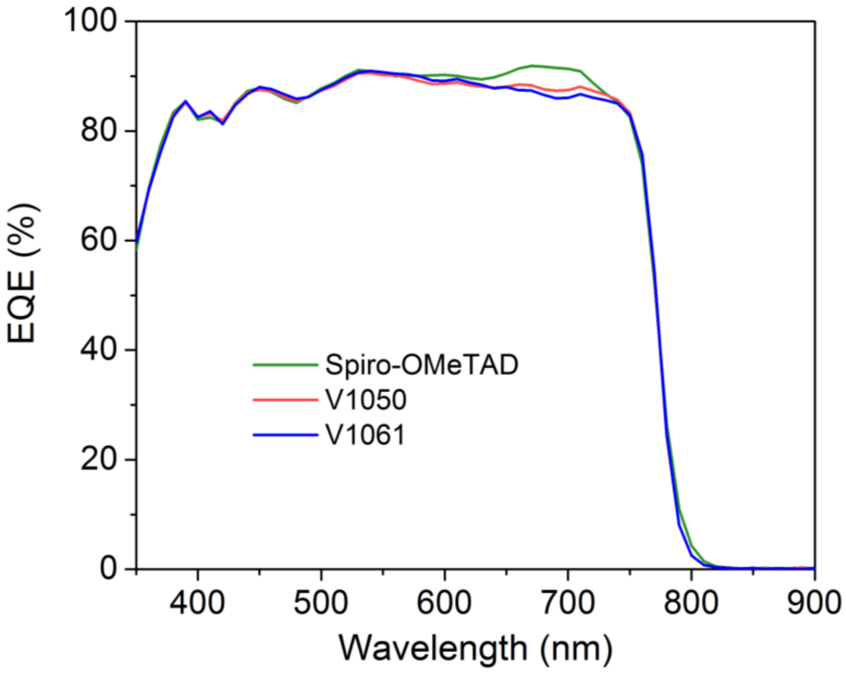

*Figure S6. External quantum efficiency of the typical PSCs parameters employing **V1050**, **V1061** and Spiro-OMeTAD*

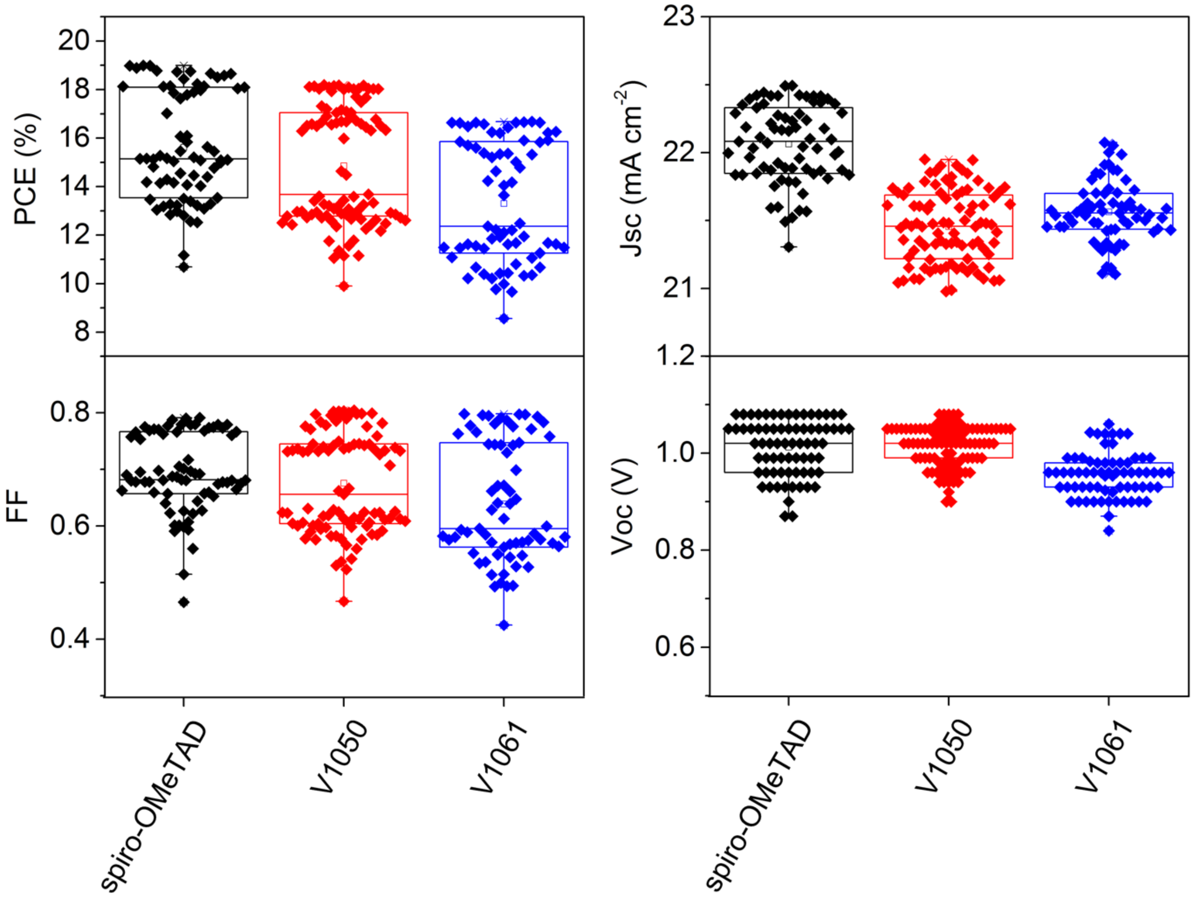

*Figure S7. The statistical distribution of the PSCs parameters employing **V1050**, **V1061** and Spiro-OMeTAD*

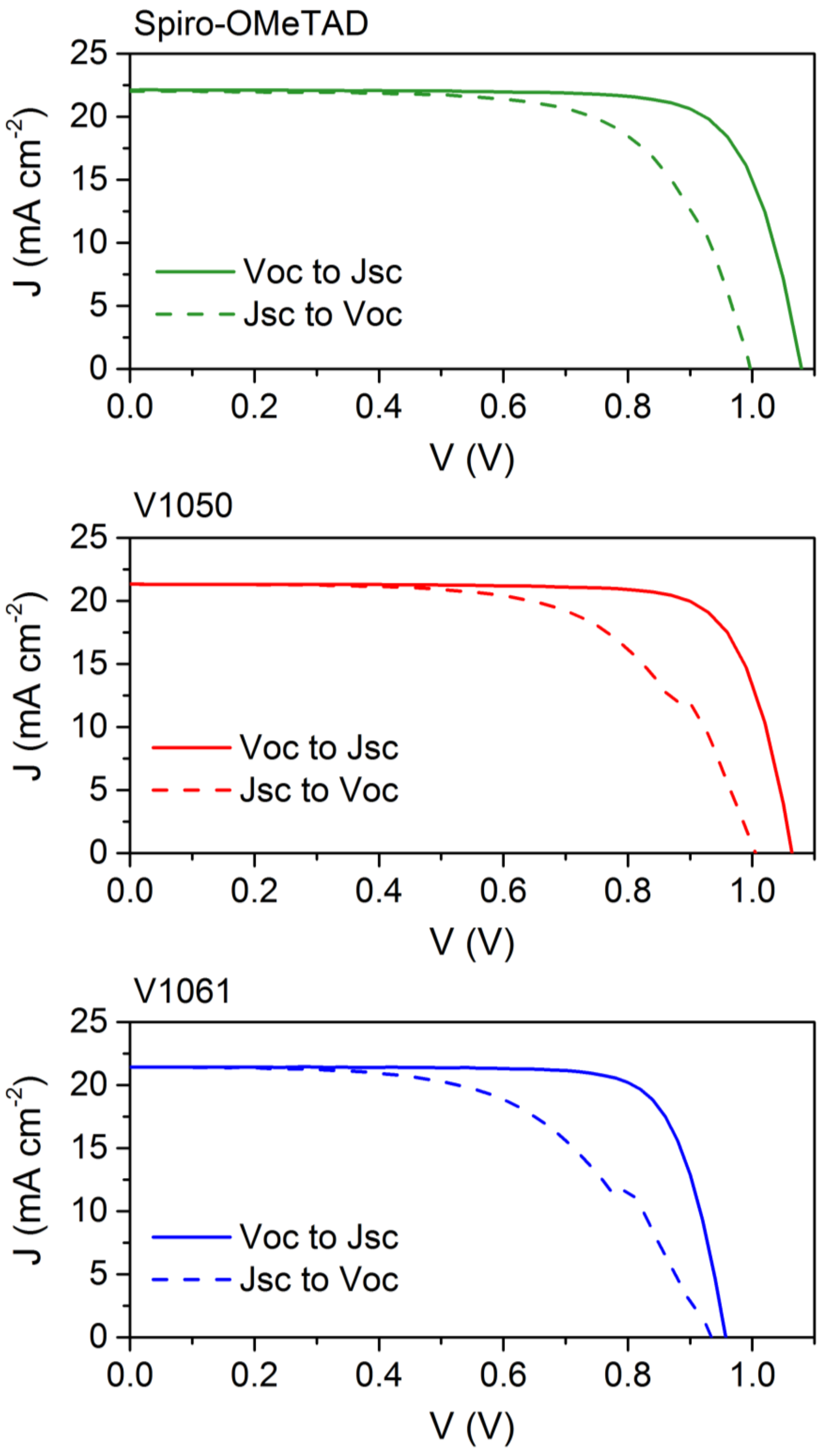

*Figure S8. JV curves from both of forward scan and reverse scan of the typical PSCs parameters employing **V1050**, **V1061** and Spiro-OMeTAD*

Contact angle measurement

Spiro-OMeTAD

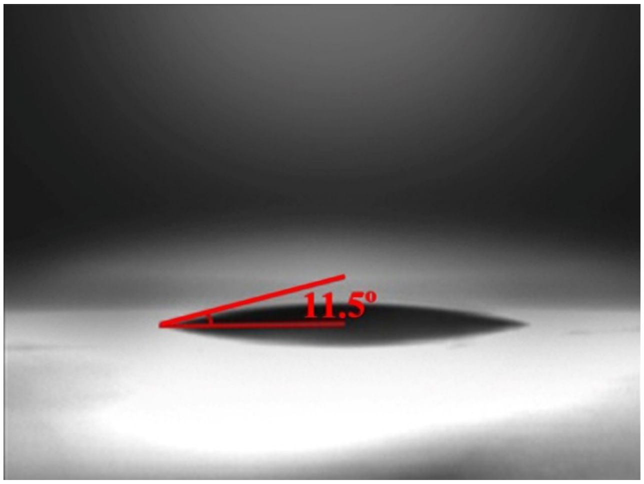

V1050

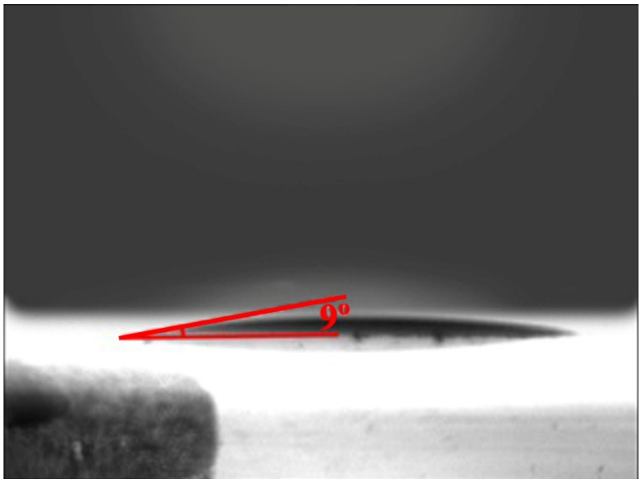

V1061

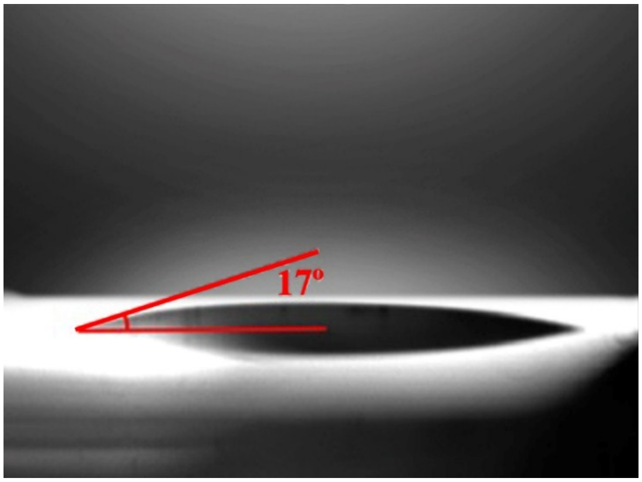

*Figure S9. Contact angle measurement of a Spiro-OMeTAD, **V1050**, and **V1061** droplet on the perovskite film. Solutions of the same concentrations and with the same additive amounts were used, as for the PSC fabrication.*

Stability of devices

To evaluate the device stability test, the devices were kept in dark at uncontrollable humidity conditions (relative humidity (RH) ~ 60 % and temperature 22°C) without encapsulation. The current–voltage (J-V) measurements and stabilized power output (SPO) measurements were measured (2400 series source meter, Keithley Instruments) under both dark and simulated solar light (AAB ABET technologies Sun 2000 solar simulator) with its light intensity, 100 mW cm^{-2} (AM 1.5) for each point. The cell aperture area of light incidence was set to 0.0913 cm^2photoactive area by employing an opaque mask.

References

1. S. Yao, K. D. Belfield, *J. Org. Chem*. **2005**, 70, 5126-5132.
2. E. Montrimas, V. Gaidelis and A. Pazera, *Lith. J. Phys*, **1966**, 6, 569-578.
3. S. M. Vaezi-Nejad, *Int. J. Electron. Theor*. **1987**, 62(3), 361-384.
4. Y. Archie, C. Chan and C. Juhasz, *Int. J. Electron*., **1987,** 62, 625–632

5. M. Kirkus, M.-H. Tsai, J. V. Grazulevicius, C.-C. Wu, L.-C. Chi and K.-T. Wong, *Synth. Met.*, **2009**, *159*, 729–734.
6. E. Miyamoto, Y. Yamaguchi and M. Yokoyama, *Electrophotography*, **1989**, *28*, 364-370.
7. M. Cardona and L. Ley, Eds., *Photoemission in Solids I*, Springer Berlin Heidelberg, Berlin, Heidelberg, **1978**, vol. 26.
